# Reduced Biofilm Accumulation on Implants Treated With Implantoplasty—An In Situ Trial With a Within‐Subject Comparison

**DOI:** 10.1002/cre2.70043

**Published:** 2024-11-28

**Authors:** Kristina Bertl, Mohammad Al‐Said, Ahmed Mourad, Magdalena Mayol, Zita Lopes da Silva, Evaggelia Papia, Andreas Stavropoulos

**Affiliations:** ^1^ Department of Periodontology, Dental Clinic, Faculty of Medicine Sigmund Freud University Vienna Austria; ^2^ Department of Periodontology Blekinge Hospital Karlskrona Sweden; ^3^ Periodontology, Faculty of Odontology Malmö University Malmö Sweden; ^4^ Department of Periodontology, Faculty of Odontology University of the Republic Montevideo Uruguay; ^5^ ETEP Research Group, Faculty of Odontology University Complutense of Madrid Madrid Spain; ^6^ Department of Oral Biology, Division 1, Faculty of Odontology Malmö University Malmö Sweden; ^7^ Department of Materials Science and Technology, Division 2, Faculty of Odontology Malmö University Malmö Sweden; ^8^ Division of Conservative Dentistry and Periodontology University Clinic of Dentistry, Medical University of Vienna Vienna Austria; ^9^ Department of Periodontology, School of Dental Medicine University of Bern Bern Switzerland

**Keywords:** biofilm, crystal violet assay, implant surface, implantoplasty, peri‐implantitis

## Abstract

**Objectives:**

This study aimed to evaluate potential differences in biofilm accumulation on three different implant surfaces: turned surface (TS), modified surface (MS), and modified surface treated with implantoplasty (IPS), using a within‐subject comparison.

**Material and Methods:**

Ten volunteers wore individualized splints containing three titanium implants with different surfaces (TS, MS, and IPS) on each buccal side of the splint. The implant position (anterior, central, and posterior) was randomly assigned among the three implants on each side. Volunteers were instructed to wear the splint for 72 h and to remove it only for eating, drinking, and performing standard oral hygiene; the splint itself was not cleaned. After 72 h, the implants were carefully removed from the splint, and the accumulated biofilm was assessed using a crystal violet assay by measuring intensity/absorbance at 570 nm.

**Results:**

All volunteers reported no deviations from the instructions. The lowest mean amount of biofilm (0.405 ± 0.07) was detected on implants of the IPS group, followed by implants of the MS (0.463 ± 0.06) and TS group (0.467 ± 0.07). A multilevel mixed‐effects linear regression analysis confirmed that implants of the IPS group accumulated a significantly lower amount of biofilm than the other surfaces (*p* < 0.001); however, no significant difference was detected between implants of the TS and MS groups (*p* = 0.806).

**Conclusions:**

Implantoplasty can generate a surface significantly less conducive to biofilm accumulation in the short term compared to pristine implants with turned or modified surfaces.

**Trial Registration**: clinicaltrials.gov identifier: NCT06049121.

## 1 Introduction

The treatment of dental implants affected by peri‐implantitis poses a clinical challenge, as in more than half of the cases, surgery is needed (Ramanauskaite, Fretwurst, and Schwarz 2021; Herrera et al. 2023). Commonly, peri‐implant bone defects have challenging morphologies; pure horizontal bone loss (i.e., a supracrestal defect) can be detected in about one out of five implants affected by peri‐implantitis, and > 50% of the implants affected by peri‐implantitis exhibit buccal or buccal and lingual bone dehiscence alongside intra‐osseous defects (Wehner et al. 2021). One of the various surgical approaches proposed for the management of such complex defects involves the mechanical removal of the threads and structured/roughened surface of implants with modified surfaces. This procedure, called implantoplasty, should be performed at those aspects of the defect where (1) the potential for bone regeneration is limited, and (2) there is a high chance for the modified implant surface to become exposed to the oral environment after treatment. With implantoplasty, the implant surface becomes smooth, thereby making it less prone to plaque/biofilm accumulation. Additionally, an implant surface after implantoplasty is easier to clean in case of exposure to the oral environment, in contrast to an exposed implant with a modified surface (Bertl and Stavropoulos 2021; Ramanauskaite and Schwarz 2024). Indeed, it is known that modified implant surfaces accumulate faster/more biofilm compared to turned implant surfaces (Quirynen et al. 1996; Teughels et al. 2006). Implantoplasty was introduced more than 30 years ago (Lozada et al. 1990), and its clinical efficacy as an adjunct to surgical peri‐implantitis treatment has already been demonstrated almost 20 years ago (Romeo et al. 2005; Romeo et al. 2007). In this clinical trial, implants treated with a non‐reconstructive approach including implantoplasty showed a survival rate of 100% and no further marginal bone loss up to 3 years after surgery, whereas implants treated likewise but without implantoplasty showed extensive progressive marginal bone loss within 2 years post‐surgery. More recent studies have confirmed good long‐term stability of the largely improved clinical outcomes after implantoplasty (Bianchini et al. 2019; Bianchini et al. 2024). Furthermore, concerns raised earlier regarding potential increased risks for mechanical (e.g., implant fracture) or biological complications (e.g., peri‐implant bone necrosis due to overheating or exacerbation of inflammation due to titanium debris) after implantoplasty are considered unfounded (Stavropoulos et al. 2019). Indeed, recent laboratory studies have indicated that only single narrow‐diameter implants with a specific design and/or with extensive bone loss (≥ 50% bone loss of the implant length) may have an increased fracture risk after implantoplasty (Bertl et al. 2021; Stavropoulos et al. 2023).

As mentioned earlier, the rationale of implantoplasty is to create a smooth implant surface that—if exposed to the oral environment—is less prone to plaque/biofilm accumulation and is easier to clean, compared with an exposed modified implant surface (Bertl and Stavropoulos 2021; Ramanauskaite and Schwarz 2024). However, there is only one study supporting this claim (Azzola et al. 2020). In this study, a single participant wore for 5 days an intra‐oral splint carrying implants with a modified surface, some of which were previously subjected to implantoplasty. Biofilm accumulation was significantly higher on the implants with a modified surface compared to implants treated with implantoplasty, with 65% and 16% of the implant surface covered with biofilm, respectively. Hence, the present in situ trial with a within‐subject comparison aimed to evaluate potential differences in biofilm accumulation among three different implant surfaces—turned surface (TS), modified surface (MS), and modified surface treated with implantoplasty (IPS)—using a larger number of volunteers compared to the previous pilot study, with the hypothesis that implantoplasty reduces the amount of biofilm accumulation.

## 2 Methods

### 2.1 Study Design and Population

The protocol of the present study was approved by the Institutional Ethics Committee (2022‐2270) and registered at clinicatltrials.gov (NCT06049121). Per protocol, 10 volunteers were recruited at the Faculty of Odontology (Malmö University, Sweden) following certain eligibility criteria: (1) ≥ 18 years of age, (2) systemically healthy, (3) no current pregnancy or breastfeeding, (4) no heavy smokers (i.e., ≤ 10 cigarettes per day), (5) no antibiotic intake in the preceding 3 months, (6) healthy periodontal conditions (Chapple et al. 2018), (7) no orthodontic appliances in the upper jaw, (8) no removable prosthesis in the upper jaw, (9) no extensive implant‐supported restorations in the upper jaw, and (10) no active carious lesions. All volunteers received information on the study purpose and associated risks and provided written informed consent before active participation.

### 2.2 Implant Material and Preparation

Sixty purpose‐made, parallel‐walled titanium implants (7 mm in length, 3.25 mm in diameter at the implant body, and 3.75 mm in diameter including the threads; Elos Medtech Dental, Gørløse, Denmark) were used for this study. Of these, 20 implants had a TS, and 40 implants had a modified surface (i.e., moderately rough, obtained by sandblasting and acid‐etching). Twenty implants with a modified surface were subjected to implantoplasty along the whole implant surface using a tungsten carbide bur sequence (i.e., two tungsten carbide burs with standard [red ring] and extra‐fine [white ring] toothing; Komet Dental, Lemgo, Germany) until a smooth surface topography was achieved. This resulted in three implant groups: (1) implants with a turned surface (TS group), (2) implants with an untreated modified surface (MS group), and (3) implants with a modified surface treated with implantoplasty (IPS group).

### 2.3 Splint Preparation

**Figure 1:**
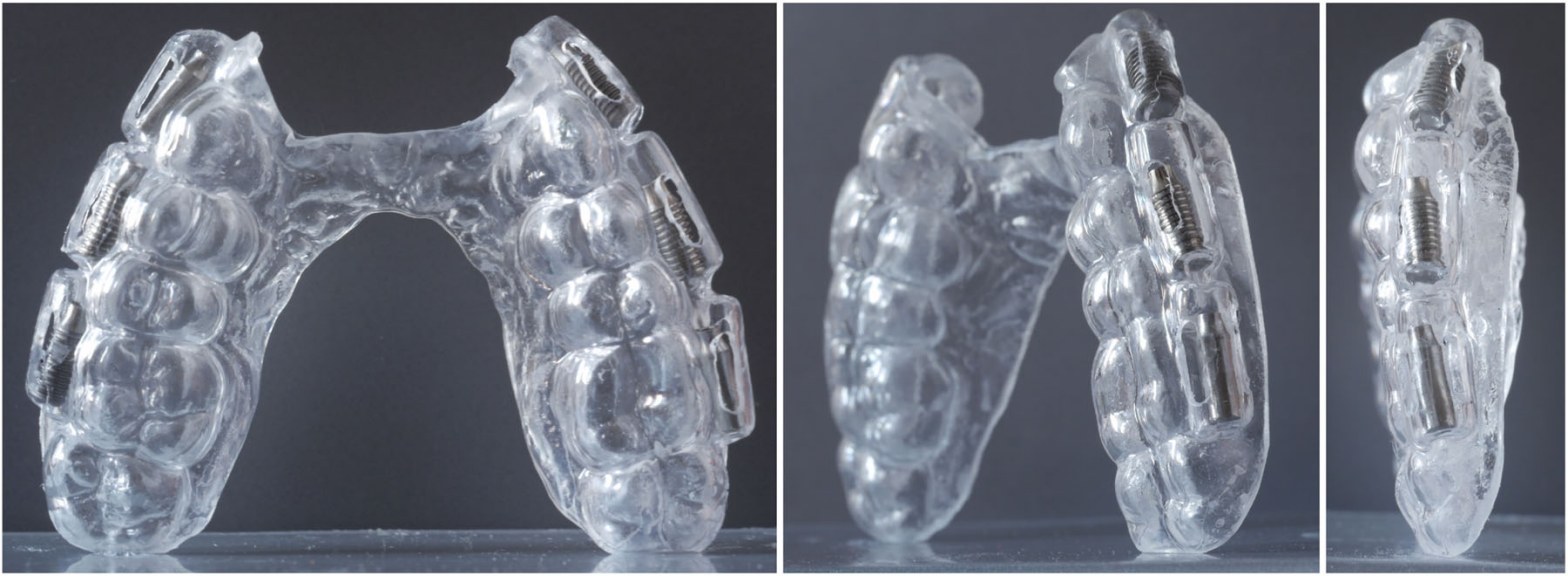
Individualized splint carrying six implants in total (i.e., one from each implant group on each side of the splint).

An alginate impression was taken from the maxilla of each volunteer, and a gypsum cast was produced. An ESSIX guide with 1 mm thickness (Biolon Klar 1.0 × 120 mm, polyethylene terephthalate glycol; Dreve Dentamid GmbH, Unna, Germany) was vacuumed on the cast. Three plastic cylinders, 10 mm in length and 4 mm in diameter, were fixed with wax at the buccal aspect of the splint in the canine, premolar, and molar regions on each side, corresponding to the future implant positions, and a second ESSIX splint with 1 mm thickness was vacuumed on top. Thus, three buccal protrusions were generated on each side of the buccal aspect of the second (outer) splint, to house the implants. The splints were then separated, the plastic cylinders were discarded, and two rectangular windows (2 × 6 mm) were created at the buccal protrusions to allow salivary flow and plaque/biofilm accumulation, and simultaneously to avoid any cleaning effect from the neighboring tissues. One implant from each of the three groups was inserted in the buccal protrusion of the second splint, on each side, according to a randomization list, which ensured an even distribution of the 20 implants of each of the three groups in the anterior (canine region), central (premolar region), and posterior (molar region) positions. Finally, the two splints were re‐assembled, glued together, and trimmed to fit without great discomfort (Figure 1).

### 2.4 In Situ Biofilm Accumulation

The volunteers were instructed to wear the splint continuously for 72 h to allow biofilm accumulation. They received a leaflet with the following instructions: (1) remove the splint for eating and drinking (except for drinking water), (2) remove the splint for performing your daily oral hygiene, (3) do not use any rinsing solution, (4) do not clean the splint in any way, and (5) store the splint in bottled water while eating/drinking or during oral hygiene; this timeframe should be as short as possible. Each participant received a bottle of water and a container for storing the splint. After 72 h, the splints were returned and carefully cut to allow removal of the implants. The implants were picked up with sterile tweezers from their upper ends, without touching the body of the implant, and inserted into sterile tubes. Biofilm accumulation was immediately analyzed.

### 2.5 Quantitative Assessment of the Accumulated Biofilm

The accumulated biofilm on the implant surface was quantified by the crystal violet assay. Each implant was rinsed three times with sterile phosphate‐buffered saline (PBS) to remove any unbound cells/material. Thereafter, the biofilm was fixated by incubation in 100% ethanol at room temperature for 30 min. After removing the fixative, the implants were dried at 37°C for 30 min with the lid of the tubes open. After another three rounds of rinsing with PBS, the biofilm on the implant surface was stained with 0.2% crystal violet solution and incubated at room temperature for 5 min. The crystal violet solution was removed, and the implants were again rinsed three times with PBS. Acetic acid (33%) was added to dissolve the bound crystal violet. Finally, 0.2 mL of this solution was transferred to a well of a 96‐well plate; this step was repeated to generate duplicate measurements of each implant. The 96‐well plate was placed in a spectrophotometer and intensity/absorbance was measured at 570 nm. The mean value of the duplicates was calculated and used for statistical analysis.

### 2.6 Sample Size Calculation

The only previous study with a similar study design that assessed implantoplasty specifically performed on implant bodies (Azzola et al. 2020) included only one participant with a large difference in biofilm accumulation between test and control implants. Based on the data provided in this specific study, a sample size calculation for comparing paired differences was performed, which resulted in the need for a single participant to confirm these differences. In order not to repeat a similar study with a single participant, it was simply decided to include 10 participants in the present study.

### 2.7 Statistical Analysis

The frequency distribution of gender, mean age, and standard deviation were calculated. The normality of the mean biofilm values was confirmed using the Shapiro–Wilk test. A multilevel mixed‐effects linear regression analysis was conducted for all implants, taking into account the fact that each volunteer contributed six implants. The amount of biofilm was defined as the primary outcome parameter, the implant group (i.e., TS, MS, and IPS) as the main predictor, and the analysis was corrected for gender and age. The first analysis used TS as the reference group, and a second analysis used MS as the reference group, to compare all three implant groups with each other. Furthermore, it was tested and confirmed that the residuals were normally distributed. Finally, an exploratory analysis (multilevel mixed‐effects linear regression analysis) was performed with the amount of biofilm as the primary outcome parameter, the implant group (i.e., TS, MS, and IPS) as the main predictor, and randomized implant position (i.e., anterior vs. central vs. posterior); the analysis was corrected for gender and age (see the Appendix). Statistical analysis was conducted using STATA/IC 17.0 for Mac, and a *p* value of ≤ 0.05 was considered statistically significant.

## 3 Results

**Figure 2:**
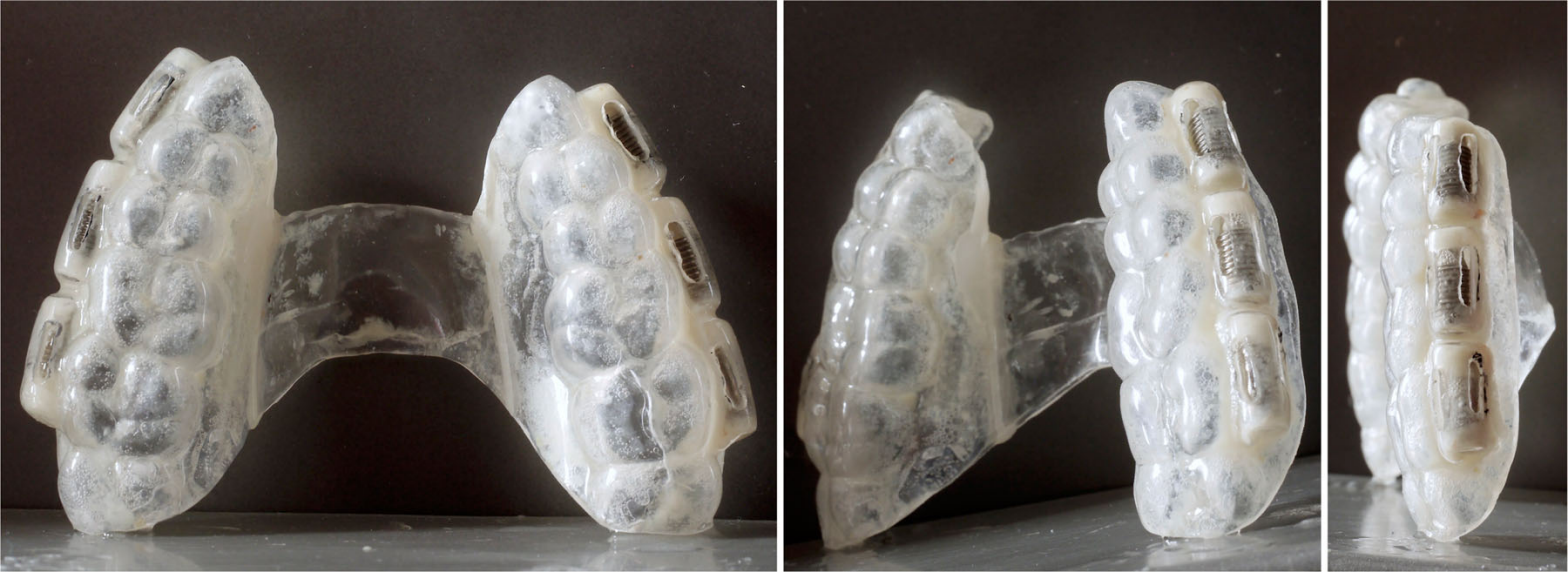
Individualized splint after 72 h of intra‐oral use. Ample accumulation of plaque/biofilm on the implants is clearly visible.

All 10 volunteers (four females and six males; mean age: 28.8 ± 11.1 years; all non‐smokers) concluded the study as planned, without any deviations or complications (Figure 2). All splints and implants were included in the analysis.

**Figure 3:**
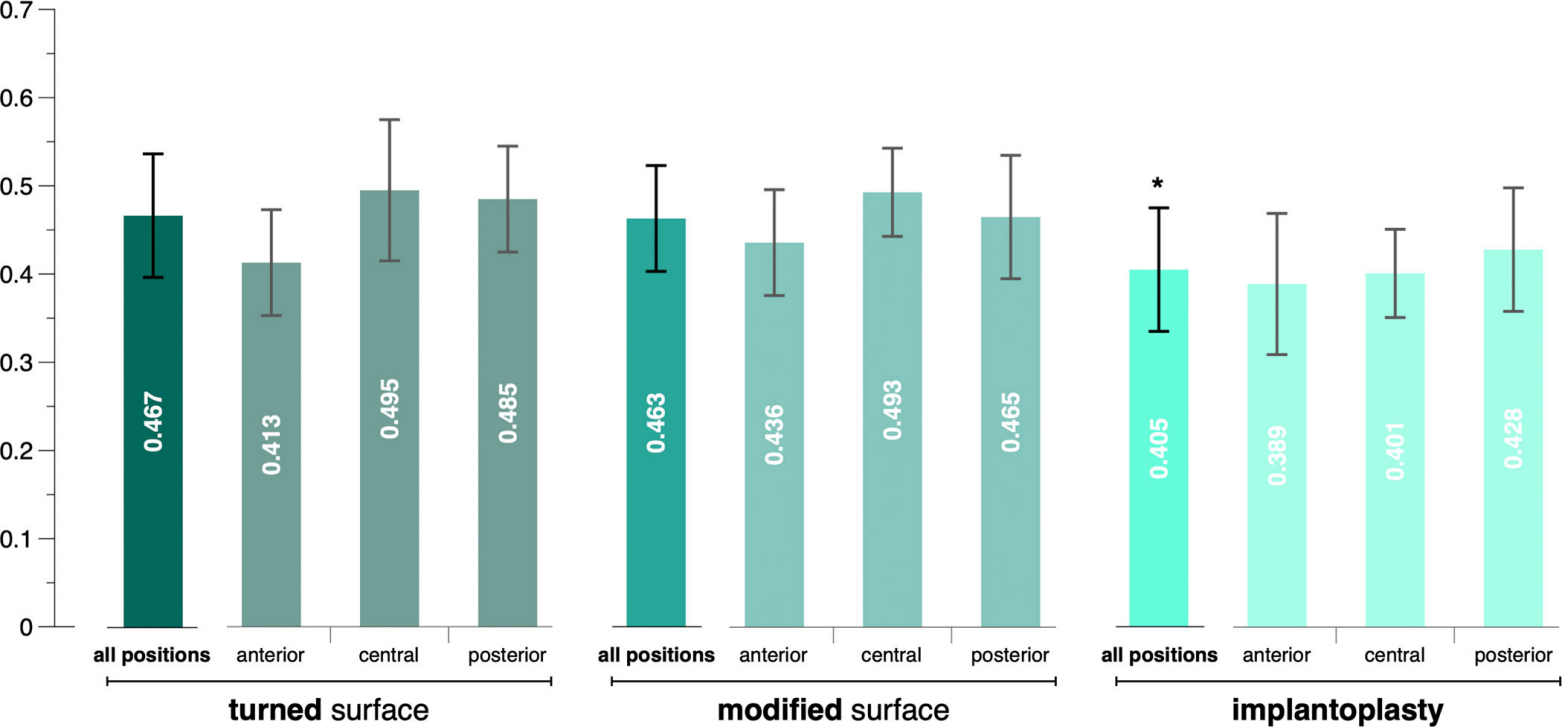
Bar graph of mean (shown in white on each bar) and standard deviation (whiskers) for biofilm accumulation, quantified using a crystal violet assay with absorbance measured at 570 nm. Data are presented separately for the three implant groups, showing either all implants of each group (“all positions”) or each implant position within the splint (“anterior,” “central,” and “posterior”). *Significantly lower compared to “all positions” of the turned and modified surface implants.

The lowest mean amount of biofilm (0.405 ± 0.07) was detected on implants of the IPS group, followed by implants of the MS (0.463 ± 0.06) and TS group (0.467 ± 0.07) (Figure 3). When comparing the three implants of each side of the splint with each other, implants of the IPS group showed the least accumulated biofilm in 13 out of 20 comparisons (i.e., 2 sides × 10 splints), whereas implants of the TS and MS groups showed the least accumulated biofilm in four and three times, respectively. The multilevel mixed‐effects linear regression analysis confirmed that implants of the IPS group accumulated a significantly lower amount of biofilm after 3 days of intra‐oral use compared to those of the other groups (*p* < 0.001); no significant difference was detected between implants of the TS and MS groups (*p* = 0.806). Furthermore, age had no significant effect on the amount of accumulated biofilm; however, females accumulated significantly more biofilm than males (*p* = 0.001) (Table 1). An exploratory analysis confirmed that biofilm accumulation at implants in the anterior position was significantly lower compared to the implants in the central and posterior positions; for details see Appendix Table A1. Furthermore, in five of seven times, where an implant of the IPS group did not show the lowest amount of biofilm accumulation compared to the pristine implants, the lowest biofilm accumulation value was observed at the implant in the anterior position.

**Table 1:** Multilevel mixed‐effects linear regression analysis with the amount of biofilm accumulation as the primary outcome parameter and implant group as the main predictor; the analysis was corrected for gender and age. A negative coefficient indicates a lower amount of biofilm accumulation.

Parameter		Coefficient	95% confidence interval		*p* value
			Lower	Upper	
Implant group^a^	TS	Ref.			
	MS	−0.004	−0.035	0.027	0.806
	IPS	**−0.062**	**−0.093**	**−0.031**	**< 0.001**
Implant group^b^	MS	Ref.			
	TS	0.004	−0.027	0.035	0.806
	IPS	**−0.058**	**−0.089**	**−0.027**	**< 0.001**
Gender	Female	Ref.			
	Male	**−0.071**	**−0.113**	**−0.030**	**0.001**
Age	Years	0.001	−0.001	0.002	0.627

Abbreviations: IPS, modified surface treated with implantoplasty; MS, modified surface; Ref., reference group; TS, turned surface.

Bold values indicate statistical significance.

^a^ TS was used as the reference group and compared with MS and IPS.

^b^ MS was used as the reference group and compared with TS and IPS.

## 4 Discussion

The results of the present study showed that biofilm accumulation, over 72 h, is significantly reduced on implants treated with implantoplasty compared to implants with turned or modified (minimally rough) surfaces, which confirmed the tested hypothesis.

As already mentioned, implant surface characteristics (i.e., roughness and topography) influence plaque/biofilm accumulation, and in general the rougher and more complex modified implant surfaces accumulate faster and/or more biofilm compared to the smoother and simpler turned implant surfaces. In particular, an arithmetic mean surface roughness (*R*_a_) < 0.2 μm is considered the “threshold” above which microbial colonization/biofilm formation significantly increases (Quirynen and Bollen 1995; Quirynen et al. 1996; Teughels et al. 2006). Although no surface measurement was performed in the present study, the observed reduced biofilm formation on implants treated with implantoplasty should be attributed to the fact that the implant surface generated by implantoplasty with a dedicated set of tungsten carbide burs has indeed a *R*_a_ value around the aforementioned threshold value. This was demonstrated in a recent laboratory study by our group; specifically, in that study, the generated surface had roughness values similar to or even lower than those of a turned surface (Yildiz, Bertl, and Stavropoulos 2022). The results herein confirm what was observed previously in a single case report, in which the test person wore a splint mounted with implants with a modified implant surface and implants treated with implantoplasty; biofilm accumulation was assessed daily for 5 days (Azzola et al. 2020). In this study, a relevant difference in favor of the implants treated with implantoplasty (i.e., much less biofilm accumulation) was detected already after 2 days of wearing the splint, and at Day 5, biofilm formation on implants with and without implantoplasty amounted, on average, to 16% and 65%, respectively (Azzola et al. 2020). Similarly, in another single case report, in vitro bacterial growth on an explanted implant—originally with a modified surface but previously treated with implantoplasty—was significantly reduced compared to that on another explanted implant—of the same brand—but previously treated with simple debridement (Geremias et al. 2017). Nevertheless, it must be mentioned that implantoplasty may not necessarily lead to surface values below/around the *R*_a_ < 0.2 μm threshold. This was reported in a relatively recent systematic review, in which *R*_a_ values between 0.3 and 0.4 μm were obtained in the original studies with different drilling/implantoplasty protocols (Burgueño‐Barris et al. 2021). The roughness of the surface after implantoplasty depends not only on the type of mechanical instruments used per se but most likely also on clinical aspects that may influence proper access to the implant surface, such as bone defect configuration and localization (e.g., lingual vs. buccal dehiscence), region in the mouth (e.g., posterior vs. anterior), possibility to remove the restoration (i.e., cemented vs. screw‐retained), and so on, together with the skills of the clinician. However, there is only limited information in the literature, about the possible impact of such factors on implant surface roughness after implantoplasty (Burgueño‐Barris et al. 2023).

In the present study, somehow unexpectedly, modified and turned surfaced implants showed a comparable amount of biofilm accumulation. This finding is likely explained by the fact that, with the current splint design, there was no “self‐cleaning” effect from surrounding tissues (e.g., cheek and tongue); the little space around the implants within the protrusions of the splint may also have limited any effective spooling effect of the saliva. Nevertheless, the finding that implants subjected to implantoplasty showed less biofilm compared to turned implants, despite being exposed to the same conditions within the splint and the similar surface roughness, may be explained by the fact that pristine turned implants simply provide more surface for biofilm accumulation due to the threading. Indeed, although implantoplasty is in general suggested for implants with a modified implant surface, the relative contribution of thread design to plaque/biofilm accumulation and/or retention is not known. This aspect may be relevant to assess in future studies, also when considering the increasing presence in the market of implants with so‐called aggressive (wider) threads.

In this context, some aspects of the present study's design should also be briefly mentioned. Herein, a standard quantitative method for plaque/biofilm accumulation has been applied, i.e., crystal violet assay (Ramachandra et al. 2023). This method is considered a reliable quantitative method, because any unbound plaque or food remnants are removed before analysis, and the total biofilm amount/total biomass of the biofilm is calculated. Yet, the crystal violet assay has the drawback that it can neither discriminate between vital and dead bacteria nor report anything on the qualitative constitution of the accumulated plaque/biofilm (Ramachandra et al. 2023). Furthermore, the assessment was performed at a single short‐term timepoint (i.e., after 72 h). This timepoint was chosen not only to facilitate participants’ compliance but also as previous studies have indicated sufficient quantitative differences in biofilm accumulation on turned and modified implant surfaces, and on implants subjected to implantoplasty, already after a short period of time (Azzola et al. 2020; Herrmann et al. 2020; Meier et al. 2023). Finally, with the chosen splint design, it was not possible to assess the impact of implantoplasty on any “self‐cleaning” action due to the mechanical impact of the surrounding tissues (i.e., cheek and tongue), nor on the effectiveness of oral hygiene measures. Indeed, implantoplasty is supposed to generate a surface that is less conducive to plaque/biofilm accumulation per se and is also easier to clean, thus overall simplifying implant care both in the dental office and at home.

The present study, although including only 10 subjects, has some relevant strengths. In particular, the study had a within‐subject comparison design, thus reducing the impact of inter‐individual variability. Indeed, the crystal violet assay values range within the TS, MS, and IPS groups, which were 0.252, 0.251, and 0.225, respectively; this range corresponded to approximately 50% of the mean values, which in turn indicated a relevant inter‐individual variability. For comparison, the crystal violet assay values for the left and right sides of each volunteer—except for a single person—differed by ≤ 0.1. Furthermore, the implants of the different groups were evenly randomized among the three positions, which was proven relevant, i.e., an exploratory analysis regarding the possible impact of the position of the implant (i.e., anterior, central, and posterior) confirmed that biofilm accumulation at the anterior implant was significantly lower compared to the other positions.

In conclusion, implantoplasty generates a surface less conducive to biofilm accumulation, at least in the short term, compared to implants with turned or modified surfaces.

## Author Contributions

**Kristina Bertl:** conceptualization, data analysis, interpretation, drafting of the manuscript, organization. **Mohammad Al‐Said:** patient recruitment and follow‐up, laboratory work, interpretation, critical review of the manuscript. **Ahmed Mourad:** patient recruitment and follow‐up, laboratory work, interpretation, critical review of the manuscript. **Magdalena Mayol:** conceptualization, laboratory work, interpretation, critical review of the manuscript. **Zita Lopes da Silva:** methods development, laboratory work, interpretation, critical review of the manuscript. **Evaggelia Papia:** conceptualization, interpretation, critical review of the manuscript, organization. **Andreas Stavropoulos:** conceptualization, data analysis, interpretation, drafting of the manuscript, organization, funding.

## Ethics Statement

The protocol of the present study was approved by the Institutional Ethics Committee (2022–2270).

## Conflicts of Interest

The authors declare no conflicts of interest.

## Data Availability

Original data are available upon reasonable request.

## 1

**Table A1:** Multilevel mixed‐effects linear regression analysis with the amount of biofilm accumulation as the primary outcome parameter, implant group as the main predictor, and randomized implant position; the analysis was corrected for gender and age. A negative coefficient indicates a lower amount of biofilm accumulation.

Parameter		Coefficient	95% confidence interval		*p* value
			Lower	Upper	
Implant group	TS	Ref.			
	MS	−0.001	−0.028	0.025	0.918
	IPS	**−0.060**	**−0.087**	**−0.033**	**< 0.001**
Implant position	Anterior	Ref.			
	Central	**0.049**	**0.022**	**0.076**	**< 0.001**
	Posterior	**0.045**	**0.019**	**0.072**	**0.001**
Gender	Female	Ref.			
	Male	**−0.071**	**−0.113**	**−0.030**	**0.001**
Age	Years	0.001	−0.001	0.002	0.627

*Note:* Bold values indicate statistical significance.

Abbreviations: IPS, modified surface treated with implantoplasty; MS, modified surface; Ref., reference group; TS, turned surface.

## References

[cre270043-bib-0001] Azzola, F. , A. C. Ionescu , M. Ottobelli , et al. 2020. “Biofilm Formation on Dental Implant Surface Treated by Implantoplasty: An In Situ Study.” Dentistry Journal 8: 40. 10.3390/dj8020040. 32384621 PMC7344745

[cre270043-bib-0002] Bertl, K. , F. Isidor , P. V. von Steyern , and A. Stavropoulos . 2021. “Does Implantoplasty Affect the Failure Strength of Narrow and Regular Diameter Implants? A Laboratory Study.” Clinical Oral Investigations 25: 2203–2211. 10.1007/s00784-020-03534-8. 32893312 PMC7966130

[cre270043-bib-0003] Bertl, K. , and A. Stavropoulos . 2021. “A Mini Review on Non‐Augmentative Surgical Therapy of Peri‐Implantitis—What Is Known and What Are the Future Challenges.” Frontiers in Dental Medicine 2: 659361.

[cre270043-bib-0004] Bianchini, M. A. , M. E. Galarraga‐Vinueza , K. Apaza‐Bedoya , J. M. De Souza , R. Magini , and F. Schwarz . 2019. “Two to Six‐Year Disease Resolution and Marginal Bone Stability Rates of a Modified Resective‐Implantoplasty Therapy in 32 Peri‐Implantitis Cases.” Clinical Implant Dentistry and Related Research 21: 758–765. 10.1111/cid.12773. 30985073

[cre270043-bib-0005] Bianchini, M. A. , L. F. Kuhlkamp , F. Schwarz , and M. E. Galarraga‐Vinueza . 2024. “Clinical and Radiographic Outcomes of Resective Surgery With Adjunctive Implantoplasty Over a 6‐ to 11‐Year Follow‐Up: A Case Series.” International Journal of Periodontics & Restorative Dentistry 44: 466–476. 10.11607/prd.6756. 37655972

[cre270043-bib-0006] Burgueño‐Barris, G. , O. Camps‐Font , R. Figueiredo , and E. Valmaseda‐Castellón . 2021. “The Influence of Implantoplasty on Surface Roughness, Biofilm Formation, and Biocompatibility of Titanium Implants: A Systematic Review.” International Journal of Oral & Maxillofacial Implants 36: e111–e119. 10.11607/jomi.8785. 34157063

[cre270043-bib-0007] Burgueño‐Barris, G. , O. Camps‐Font , R. Figueiredo , and E. Valmaseda‐Castellón . 2023. “Factors Affecting Implant Surface Roughness and Platform Alterations After Implantoplasty: An In Vitro Study Simulating Different Clinical Scenarios.” International Journal of Oral & Maxillofacial Implants 38: 739–746. 10.11607/jomi.10074. 37669511

[cre270043-bib-0008] Chapple, I. L. C. , B. L. Mealey , T. E. Van Dyke , et al. 2018. “Periodontal Health and Gingival Diseases and Conditions on an Intact and a Reduced Periodontium: Consensus Report of Workgroup 1 of the 2017 World Workshop on the Classification of Periodontal and Peri‐Implant Diseases and Conditions.” Journal of Clinical Periodontology 45, no. S20: S68–S77. 10.1111/jcpe.12940. 29926499

[cre270043-bib-0009] Geremias, T. C. , J. F. D. Montero , R. S. Magini , G. Schuldt Filho , E. B. de Magalhães , and M. A. Bianchini . 2017. “Biofilm Analysis of Retrieved Dental Implants After Different Peri‐Implantitis Treatments.” Case Reports in Dentistry 2017: 8562050. 10.1155/2017/8562050. 28487780 PMC5401748

[cre270043-bib-0010] Herrera, D. , T. Berglundh , F. Schwarz , et al. 2023. “Prevention and Treatment of Peri‐Implant Diseases – The EFP S3 Level Clinical Practice Guideline.” Journal of Clinical Periodontology 50, no. S26: 4–76. 10.1111/jcpe.13823. 37271498

[cre270043-bib-0011] Herrmann, H. , J. S. Kern , T. Kern , J. Lautensack , G. Conrads , and S. Wolfart . 2020. “Early and Mature Biofilm on Four Different Dental Implant Materials: An In Vivo Human Study.” Clinical Oral Implants Research 31: 1094–1104. 10.1111/clr.13656. 32871610

[cre270043-bib-0012] Lozada, J. L. , R. A. James , M. Boskovic , C. Cordova , and S. Emanuelli . 1990. “Surgical Repair of Peri‐Implant Defects.” Journal of Oral Implantology 16: 42–46. 2074590

[cre270043-bib-0013] Meier, D. , M. Astasov‐Frauenhoffer , T. Waltimo , L. K. Zaugg , N. Rohr , and N. U. Zitzmann . 2023. “Biofilm Formation on Metal Alloys and Coatings, Zirconia, and Hydroxyapatite as Implant Materials In Vivo.” Clinical Oral Implants Research 34: 1118–1126. 10.1111/clr.14146. 37489537

[cre270043-bib-0014] Quirynen, M. , C. M. Bollen , W. Papaioannou , J. Van Eldere , and D. van Steenberghe . 1996. “The Influence of Titanium Abutment Surface Roughness on Plaque Accumulation and Gingivitis: Short‐Term Observations.” International Journal of Oral & Maxillofacial Implants 11: 169–178. 8666447

[cre270043-bib-0015] Quirynen, M. , and C. M. L. Bollen . 1995. “The Influence of Surface Roughness and Surface‐Free Energy on Supra‐ and Subgingival Plaque Formation in Man. A Review of the Literature.” Journal of Clinical Periodontology 22: 1–14. 10.1111/j.1600-051x.1995.tb01765.x. 7706534

[cre270043-bib-0016] Ramachandra, S. S. , P. Wright , P. Han , A. Abdal‐hay , R. S. B. Lee , and S. Ivanovski . 2023. “Evaluating Models and Assessment Techniques for Understanding Oral Biofilm Complexity.” MicrobiologyOpen 12: e1377. 10.1002/mbo3.1377. 37642488 PMC10464519

[cre270043-bib-0017] Ramanauskaite, A. , T. Fretwurst , and F. Schwarz . 2021. “Efficacy of Alternative or Adjunctive Measures to Conventional Non‐Surgical and Surgical Treatment of Peri‐Implant Mucositis and Peri‐Implantitis: A Systematic Review and Meta‐Analysis.” International Journal of Implant Dentistry 7: 112. 10.1186/s40729-021-00388-x. 34779939 PMC8593130

[cre270043-bib-0018] Ramanauskaite, A. , and F. Schwarz . 2024. “Current Concepts for the Treatment of Peri‐Implant Disease.” International Journal of Prosthodontics 37: 124–134. 10.11607/ijp.8750. 37988432

[cre270043-bib-0019] Romeo, E. , M. Ghisolfi , N. Murgolo , M. Chiapasco , D. Lops , and G. Vogel . 2005. “Therapy of Peri‐Implantitis With Resective Surgery. A 3‐year Clinical Trial on Rough Screw‐Shaped Oral Implants. Part I: Clinical Outcome.” Clinical Oral Implants Research 16: 9–18. 10.1111/j.1600-0501.2004.01084.x. 15642026

[cre270043-bib-0020] Romeo, E. , D. Lops , M. Chiapasco , M. Ghisolfi , and G. Vogel . 2007. “Therapy of Peri‐Implantitis With Resective Surgery. A 3‐year Clinical Trial on Rough Screw‐Shaped Oral Implants. Part II: Radiographic Outcome.” Clinical Oral Implants Research 18: 179–187. 10.1111/j.1600-0501.2006.01318.x. 17348882

[cre270043-bib-0021] Stavropoulos, A. , K. Bertl , S. Eren , and K. Gotfredsen . 2019. “Mechanical and Biological Complications After Implantoplasty – A Systematic Review.” Clinical Oral Implants Research 30: 833–848. 10.1111/clr.13499. 31254417

[cre270043-bib-0022] Stavropoulos, A. , K. Bertl , F. Isidor , and P. Vult von Steyern . 2023. “Implantoplasty and the Risk of Fracture of Narrow Implants With Advanced Bone Loss: A Laboratory Study.” Clinical Oral Implants Research 34: 1038–1046. 10.1111/clr.14132. 37464268

[cre270043-bib-0023] Teughels, W. , N. Van Assche , I. Sliepen , and M. Quirynen . 2006. “Effect of Material Characteristics and/or Surface Topography on Biofilm Development.” Clinical Oral Implants Research 17, no. S2: 68–81. 10.1111/j.1600-0501.2006.01353.x. 16968383

[cre270043-bib-0024] Wehner, C. , K. Bertl , G. Durstberger , C. Arnhart , X. Rausch‐Fan , and A. Stavropoulos . 2021. “Characteristics and Frequency Distribution of Bone Defect Configurations in Peri‐Implantitis Lesions – A Series of 193 Cases.” Clinical Implant Dentistry and Related Research 23: 178–188. 10.1111/cid.12961. 33174377 PMC8246974

[cre270043-bib-0025] Yildiz, H. , K. Bertl , and A. Stavropoulos . 2022. “Titanium Implant Surface Roughness After Different Implantoplasty Protocols: A Laboratory Study.” Clinical and Experimental Dental Research 8: 1315–1321. 10.1002/cre2.659. 36069295 PMC9760168

